# Protegrin-2, a potential inhibitor for targeting SARS-CoV-2 main protease M^pro^

**DOI:** 10.1016/j.csbj.2023.07.020

**Published:** 2023-07-24

**Authors:** Zainab Jan, Anupriya M. Geethakumari, Kabir H. Biswas, Puthen Veettil Jithesh

**Affiliations:** a Division of Genomics and Translational Biomedicine, College of Health & Life Sciences, Hamad Bin Khalifa University, Education City, Qatar Foundation, Doha 34110, Qatar; b Division of Biological and Biomedical Sciences, College of Health & Life Sciences, Hamad Bin Khalifa University, Education City, Qatar Foundation, Doha 34110, Qatar

**Keywords:** M^pro^, COVID-19, SARS-CoV-2, antimicrobial peptides, BRET assay, Protegrin-2

## Abstract

**Background:**

SARS-CoV-2 variants continue to spread throughout the world and cause waves of COVID-19 infections. It is important to find effective antiviral drugs to combat SARS-CoV-2 and its variants. The main protease (M^pro^) of SARS-CoV-2 is a promising therapeutic target due to its crucial role in viral replication and its conservation in all the variants. Therefore, the aim of this work was to identify an effective inhibitor of M^pro^.

**Methods:**

We studied around 200 antimicrobial peptides using *in silico* methods including molecular docking and allergenicity and toxicity prediction. One selected antiviral peptide was studied experimentally using a Bioluminescence Resonance Energy Transfer (BRET)-based M^pro^ biosensor, which reports M^pro^ activity through a decrease in energy transfer.

**Results:**

Molecular docking identified one natural antimicrobial peptide, Protegrin-2, with high binding affinity and stable interactions with M^pro^ allosteric residues. Furthermore, free energy calculations and molecular dynamics simulation illustrated a high affinity interaction between the two. We also determined the impact of the binding of Protegrin-2 to M^pro^ using a BRET-based assay, showing that it inhibits the proteolytic cleavage activity of M^pro^.

**Conclusions:**

Our *in silico* and experimental studies identified Protegrin-2 as a potent inhibitor of M^pro^ that could be pursued further towards drug development against COVID-19 infection.

## Highlights

• The main protease (M^pro^) of SARS-CoV-2 is a promising therapeutic target.
• We studied around 200 antimicrobial peptides using *in silico* methods.
• One peptide, Protegrin-2, was studied *in vitro* using a BRET-based M^pro^ biosensor.
• Protegrin-2 is a potent inhibitor of M^pro^ hence useful against COVID-19 infection.

## Graphical Abstract

**Figure:**
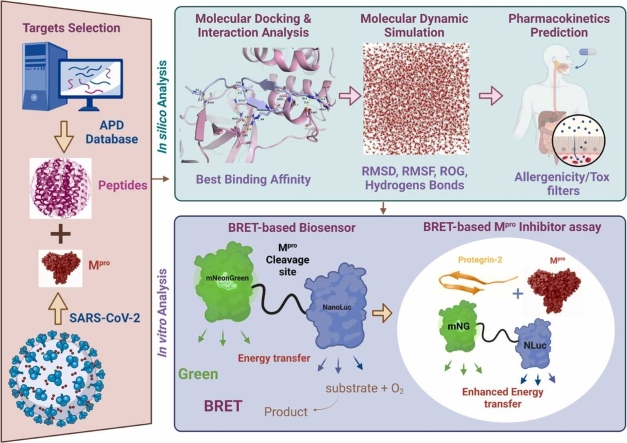

## 1 Introduction

Since the beginning of the COVID-19 pandemic, several variants have appeared with enhanced virulence, increased transmission capabilities, or potential for immune escape [1], [2]. The main protease (M^pro^, also known as 3CL protease), which is conserved among all the variants, is crucial for SARS-CoV-2 life cycle. Hence, the M^pro^ is a potential target for the development of antiviral drugs [3]. Antimicrobial peptides (AMPs) have been extensively explored for their potential antiviral activities in various infections in recent years [4], [5], [6], [7], [8]. They are biologically active molecules produced by a wide variety of organisms, including humans, as an essential component of their innate immune response against invading pathogens. AMPs are short sequence peptides ranging from 10 to 100 amino acids, positively charged and amphiphilic. The Antimicrobial Peptide Database (APD) consists of 3425 AMPs, among which 200 are antiviral [9]. The mode of action of these peptides varies, including the blockage of interactions, direct inhibition of virus, and inhibition of absorption. Some AMPs also reduce the expression of viral genes [10]. Moreover, the positively charged residues on AMPs interact with negatively charged residues on the cell surface, such as heparan sulfate, which contains glycosaminoglycans that are associated with viral attachment. AMPs can also inhibit the spread of cell-to-cell infection and formation of syncytium [4]. These peptides also have great efficacy, with minimal toxicity in humans, and hence excellent specificity and effectiveness can be achieved at low dosages with little side effects [10]. Therefore, we explored the antiviral activities of naturally occurring antiviral peptides through their ability to inhibit M^pro^. In this study, we predict that Protegrin-2 can strongly bind to M^pro^ and show it to inhibit the activity of M^pro^ *in vitro*.

## 2 Materials and methods

### 2.1 Preparation of the protein and peptides

The 3D structure of M^pro^ was downloaded from the RCSB Protein Data Bank (PDB ID: 7LKD)[11]. A total of 200 reported antiviral peptides of different species were selected from the antimicrobial peptide database (APD) [9]. The 3D structures of 62 peptides were retrieved from PDB, whereas the 3D structures of 138 peptides were designed using AlphaFold [12]. Pymol 1.7.4.5 was used for geometry optimization and minimization of all the selected peptides and the protein [13].

### 2.2 Protein-peptide docking studies

HDock server [14] was used to find the binding interactions of M^pro^ with the selected peptides. The docking prediction jobs for all the 200 peptides were submitted to the server by using the template free docking option. The best pose complexes with low binding energies were selected for the interaction analysis. For the interaction analysis and visualization, PDBsum [15] and Pymol version 1.7.4.5 [13] were used.

### 2.3 Allergenicity and toxicity prediction

AllerTOP v. 2.0 [16], AllergenFP v.1.0 [17], AllerCatPro 2.0 [18], and AlgPred 2.0 [19] webservers were used for the prediction of allergenicity of these peptides and ToxinPred was employed for the prediction of toxicity [20] using default parameters.

### 2.4 Molecular dynamics (MD) simulation

Molecular dynamics simulations of apo- and holo-M^pro^ were carried out using Gromacs version 5.1.4 [21]. Ligand topology files were created using automated topology builder (ATB). The complete protein complexes were solvated in three-dimensional box with a size of 534.76 nm^3^ along with simple point charge (SPC) water. Three Na^+^ ions were added in the solution. Steric overlap was eliminated by using sharpest descent algorithm, and each system was subjected to 5000 energy minimization steps with a cut-off > 1000 kJmol^−1^. Following that, all the systems underwent a two-stage equilibration phase called NVT and NPT. The system's temperature was stabilized for 500 picoseconds (ps) using the NVT equilibration, and the pressure was stabilized for 500 ps using NPT. The V-rescale temperature-coupling [22] method was employed for the NVT system, whereas the Nose-Hoover pressure coupling [23] was used for NPT with a temperature of 303.15 K at 1 ps. The Particle Mesh Ewald method was used for the calculation of electrostatic forces for both the NVT and NPT systems [24]. Finally, all the systems were subjected to a full 100 nanoseconds (ns) simulation. Analysis of the MD trajectory files was carried out using the GROMACS tools. The root mean square deviation (RMSD) was determined using gmx rmsd whereas the calculation for root mean square fluctuation (RMSF) was done using gmx rmsf. Moreover, the radius of gyration (Rog) and the number of hydrogen bonds were determined using gmx gyrate and gmx hbond.

### 2.5 Cell lysate preparation for *in vitro* BRET assays

HEK 293 T cells were seeded onto 10 cm dish one day prior to transfection. The cells were transfected with the plasmid encoding the BRET-based M^pro^ sensor construct (pmNG-M^pro^-Nter-auto-NLuc) [25], using 150 μg of polyethylenimine (PEI) lipid (Sigma-Aldrich; 408727–100 mL) in Opti-MEM (Invitrogen; 31985088). At 48 h post-transfection, the cells were washed with chilled Dulbecco's Phosphate-Buffered Saline (DPBS) and lysed in a buffer containing 50 mM HEPES (pH 7.5), 50 mM NaCl, 0.1 % Triton X-100, 1 mM dithiothreitol (DTT) & 1 mM ethylenediamine tetra acetic acid (EDTA) on ice, followed by centrifugation at 4 °C for 1 h at 14,000 rotations per min (RPM). The supernatant was collected, aliquoted and stored at −80 °C until further usage.

### 2.6 Expression and purification of recombinant SARS-CoV-2 M^pro^

A plasmid (pETM33_NSP5_M^pro^; a gift from Ylva Ivarsson (Addgene plasmid # 156475; http://n2t.net/addgene:156475; RRID: Addgene_156475)) encoding the SARS-CoV-2 M^pro^ was transformed into *Escherichia coli* (*E. coli*) BL21-CodonPlus cells (Agilent Technologies). The protein was expressed upon induction with 0.1 mM isopropyl‐β‐D‐thiogalactopyranoside (IPTG) at 37 °C for 2.50 h. The cells were harvested by centrifugation (4000 g, 10 min, 4 °C) and the cell pellet was resuspended in 10 mL lysis buffer containing 50 mM Tris (pH 8), 300 mM NaCl, 10 mM β-mercaptoethanol (β-ME), 1 mM PMSF and 10 % (v/v) glycerol and lysed by sonication [21], [22], [26], [27]. The supernatant was collected after centrifugation (18000 g, 90 min, 4 °C) and incubated with pre-equilibrated glutathione (GSH) beads at 4 °C for 2 h. The beads were washed with a buffer containing 50 mM Tris (pH 7), 150 mM NaCl, 10 mM β-ME, 1 mM EDTA, 0.01 % Triton X-100 % and 10 % glycerol. Following washes, the beads were incubated with the PreScission Protease (GE Healthcare #27–0843–01) in a buffer containing 50 mM Tris (pH 7), 150 mM NaCl, 1 mM DTT, 1 mM EDTA, 0.01 % Triton X-100 % and 10 % glycerol) at 4 °C for 16 h to cleave M^pro^ from the GST-tag. The supernatant containing SARS-CoV-2 M^pro^ protease was collected after centrifugation at 500 g at 4 °C for 10 min.

### 2.7 *In vitro*, BRET-based M^pro^ proteolytic cleavage inhibitor assay

Recombinantly purified SARS-CoV-2 M^pro^ protease (500 nM) was incubated with various concentrations (ranging from 10^−6^ to 10^−12^ M) of Recombinant Pig Protegrin-2 (NPG2- CSB-EP341296PI, 20 microgram) in a buffer containing Tris-buffered saline (TBS), 1 M sodium citrate, 1 mM EDTA and 2 mM DTT for 2 h at 37 °C. GC376 (GC376 Sodium; AOBIOUS - AOB36447; stock solution prepared in 50 % DMSO at a concentration of 10 mM) served as control. The cell lysate containing mNG-M^pro^-Nter-auto-NLuc sensor was added and further incubated for 2 h at 37 °C. BRET measurements were performed at 37 °C by the addition of furimazine (Promega, Wisconsin, USA) at a dilution of 1:200. The bioluminescence (467 nm) and fluorescence (533 nm) readings were recorded using Tecan SPARK multimode microplate reader and used to calculate the BRET ratios (533 nm/467 nm).

## 3 Results

### 3.1 Molecular docking of M^pro^ with antimicrobial peptides

**Fig. 1:**
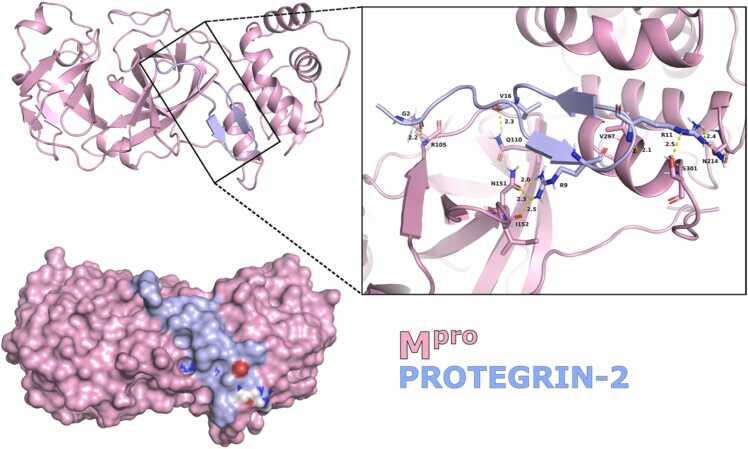
Docked complex of M^pro^ with Protegrin-2; Yellow lines in the figure on the right represent hydrogen bonds.

Docking of the M^pro^ protein with the 200 antiviral peptides present in the AMP database revealed the differences in binding affinities (Supplementary Table 1). Out of these peptides, Protegrin-2 (PDB ID: 2MUH) [28] exhibited the highest binding affinity with M^pro^ (HDock scores: −216.84 kcal/mol) (Fig. 1). Protegrin-2 showed the highest binding potential at the allosteric site, forming eight hydrogen bond interactions with the side residues (D176, Q110, F294, Y154, N151, I152 and D153) of M^pro^. Hence, we took forward Protegrin-2 for further detailed analysis as described below.

### 3.2 Allergenicity and toxicity prediction

To assess the suitability of Protegrin-2 as a candidate drug, we performed allergenicity and toxicity analysis. Protegrin-2 was characterized as probable non-allergen by three out of the four tools used for prediction (AllergenFP v.1.0 [17], AllerCatPro 2.0 [16] and AlgPred 2.0 [19]). Protegrin-2 was also predicted as a non-toxic peptide by ToxinPred [20].

### 3.3 Molecular dynamics simulation of M^pro^ with protegrin-2

**Fig. 2:**
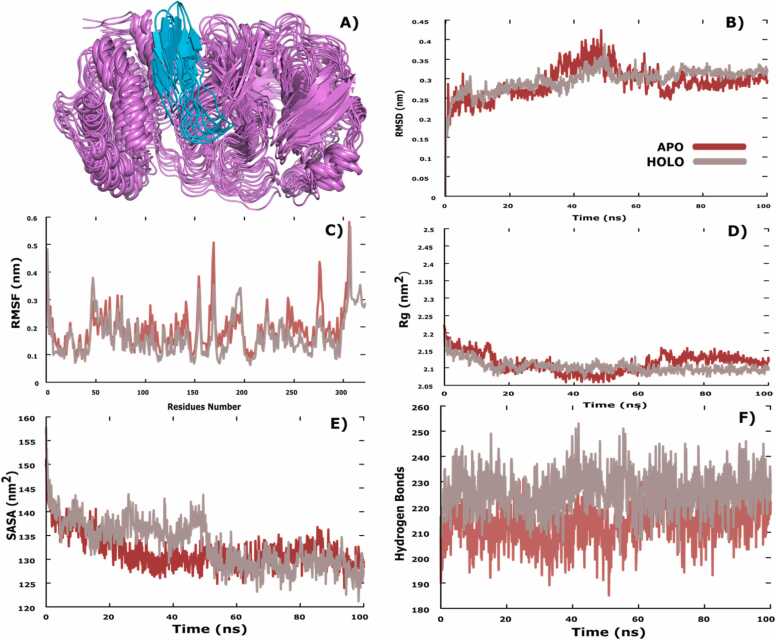
**Stable interaction of Protegrin-2 with M**^**pro**^. A. Composite image of snapshots representing 100 ns simulation, taken 10 ns apart. B. Root mean square deviation (RMSD) measurements of the backbone atoms of apo- and holo-M^pro^ throughout the 100 ns MD simulations. C. Root mean square fluctuation (RMSF) measurements of Ca atoms of residues in apo and holo-M^pro^ across 100 ns MD simulation. D. Radius of gyration (Rog) measurements of apo and holo-M^pro^ across 100 ns MD simulations. E. Solvent accessible surface area (SASA) of apo- and holo-M^pro^ across the 100 ns MD simulations. F. Number of hydrogen bonds of apo and holo-M^pro^ across the 100 ns MD simulations.

**Fig. 3:**
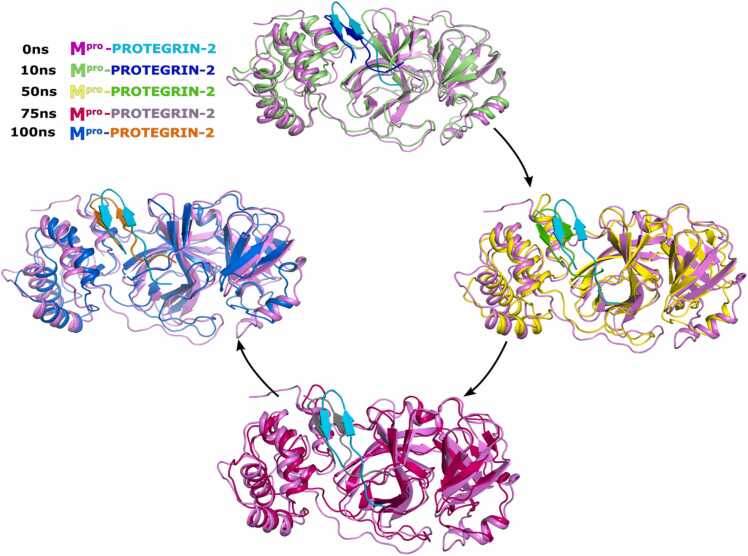
Superimposed structure of M^pro^-Protegrin-2 complex at 0 ns, 10 ns, 50 ns, 75 ns and 100 ns simulation demonstrating the structural alterations of M^pro^-Protegrin-2 complex during the 100 ns simulation.

To assess the stability of binding of Protegrin-2 with M^pro^, we performed MD simulations of the docked complex (Fig. 2A and Fig. 3). Trajectory analysis of the root-mean-square deviation (RMSD) values of the backbone indicated that the M^pro^-Protegrin-2 complex reached equilibrium during the 100 ns simulation and the low RMSD values showed a stable complex formation (Fig. 2B). The RMSD of holo-M^pro^ with Protegrin-2 between the first frame at 0 ns (F0) and the last frame at 100 ns (F100) reached 0.3 nm, and it was only 0.4 nm when compared with the frame at 50 ns (F50) to F100 (Fig. 2B). Moreover, the root-mean-square fluctuation (RMSF) revealed a region of high dynamic fluctuation in apo-M^pro^ between residues aa 50 – 150 (Fig. 2C). The radius of gyration (Rog) of M^pro^-Protegrin-2 first decreased between 0 and 20 ns and after the 20 ns increased to reach 2.15 nm followed by a sharp decrease to reach 2.1 nm at 80 ns and then stable up to 100 ns (Fig. 2D). The complex showed a high value of solvent-accessible surface area (SASA) between 0 ns and 50 ns compared to the apo-M^pro^ (Fig. 2E). The number of hydrogen bonds in the M^pro^-protegrin-2 complex increased between 0 and 100 ns as compared to apo-M^pro^ (Fig. 2F). Importantly, we found a sustained H-bond between Protegrin-2 and the Q110 residue in M^pro^ with an occupancy of 70.68 %, whereas the residue N151 of M^pro^ formed hydrogen bonds with Protegrin-2 with occupancies of 73.26 %.

### 3.4 Inhibition of M^pro^ activity with protegrin-2

**Fig. 4:**
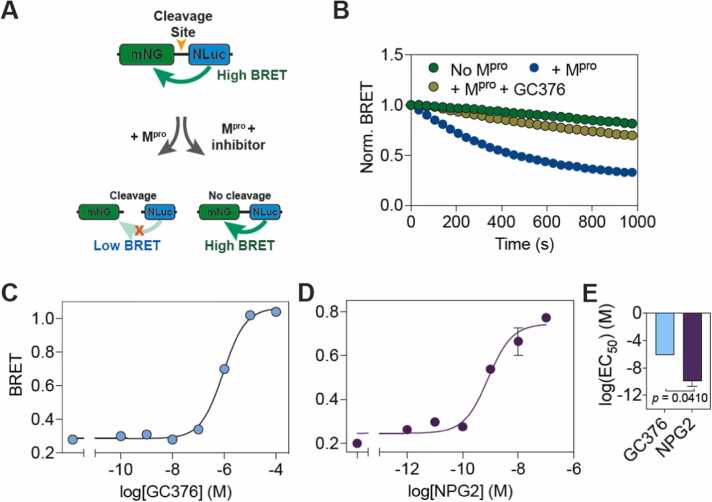
**Protegrin-2 inhibits M**^**pro**^***in vitro***. (A) Schematic illustration of the BRET-based M^pro^ sensor. (B) Graph showing BRET ratio of the M^pro^ sensor in the absence of M^pro^, in the presence of M^pro^ and in the presence of M^pro^ treated with inhibitor GC376 (100 μM). (C, D) Graphs showing BRET of the M^pro^ sensor upon incubation with the indicated concentrations of GC376 (C) and protegrin-2 (D). (E) Graph comparing the EC_50_ values of GC376 and protegrin-2. Data shown are mean ± s.e.m obtained from two independent experiments.

Based on our computational results discussed above, we proceeded to determine the impact of Protegrin-2 on the catalytic activity of M^pro^. The inhibitory potential of Protegrin-2 on the catalytic activity of the M^pro^ was determined by *in vitro* assays using an M^pro^ sensor that we recently developed [29] (Fig. 4**A**). This sensor is based on bioluminescence resonance energy transfer (BRET), wherein the efficiency of energy transfer between a donor and acceptor largely depends on the distance between the two proteins [29], [30], [31], [32], [33], [34]. The sensor consists of a green fluorescent protein, mNeonGreen, that serves as the resonance energy acceptor and a bioluminescent protein, NanoLuc, that serves as the energy donor sandwiching the cognate M^pro^ N-terminal autocleavage site. In the absence of M^pro^-mediated cleavage of the sensor, a high resonance energy transfer is observed while M^pro^-mediated cleavage of the peptide results in a substantial decrease in resonance energy transfer, as determined from the ratio of emission at 533 nm (mNeonGreen emission) and 467 nm (NLuc emission). For this, we expressed the M^pro^ sensor in HEK 293 T cells and prepared cell lysates while SARS-CoV-2 M^pro^ was expressed and purified from bacteria. Incubation of the sensor with the recombinantly purified M^pro^ resulted in a decrease in BRET, while inclusion of the known M^pro^ inhibitor, GC376 (100 μM), resulted in the abrogation of the decrease in BRET (Fig. 4**B**). Further, incubation with a range of GC376 concentrations resulted in a dose-dependent decrease in M^pro^ proteolytic activity as indicated by the increase in BRET (Fig. 4**C**), with an IC_50_ value (0.9 ± 0.1 μM) similar to that reported previously [47–51]. We then performed dose-response experiments with Protegrin-2 and found the peptide to inhibit M^pro^ with high affinity, with an IC_50_ values of 0.83 ± 0.07 nM as compared to GC376 (Fig. 4**D,E**).

## 4 Discussion

We have identified Protegrin-2 as a potential inhibitor of the SARS-CoV-2 main protease M^pro^ through large-scale *in silico* and focused *in vitro* studies. COVID-19 reached pandemic proportions due to the emergence of SARS-CoV-2 variants such as B.1.1.7, B.1.1.529, BA.2 with increased transmissibility and the ability to evade immune response against the spike (S) glycoprotein, which helps in the virus entry to the host cell. The main protease M^pro^, which is largely conserved among several SARS-CoV-2 variants, provides an alternate target for inhibition of the virus [35]. Naturally occurring antimicrobial peptides derived from various sources have been found to be effective against viruses and a few against SARS-CoV-2 as well, including LL-37 (cathelicidin) [36], protegrin-1 [36], P9R [37], beta-defensin 1 [36], human intestine defensin-5 [38], [39].

High selectivity, safety, tolerability, and efficacy of peptides have attracted the interest of researchers in developing peptide-based treatments [40], [41]. The identification of anti-SARS-CoV-2 activity in numerous naturally and synthetically created peptides targeting viral attachment and replication reinforces the need for peptide-based prophylactics and treatment against SARS-CoV-2. However, there are also potential downsides to peptide-based treatments, such as chemical and physical instability, sensitivity to proteolytic hydrolysis, a tendency for aggregation, and limited bioavailability and membrane permeability [42], [43]. Several ways of overcoming these constraints have been proposed, such as the modification of both the side chains and the amide bond which can result in proteolytically resistant peptidomimetics. Moreover, the incorporation of D-amino acids into the peptide causes cyclization, which confers resistance to proteolytic breakdown and enhances absorption following oral administration [40], [42]. Such methodologies can aid in the development of safe, efficient, and effective peptide-based vaccines and therapies.

Protegrin-2 (NPG-2) is a cysteine rich potent antimicrobial peptide originally isolated from pigs [44]. Two cysteine-cysteine disulfide bridges serve to stabilize the anti-parallel hairpin conformation of Protegrin-2, which greatly enhances its antimicrobial activity [45]. This cyclic peptide has low toxicity, and the conformational rigidity and increased surface area of cyclic structures contribute to their high selectivity and increased affinity [46]. Furthermore, our BRET-based assay provided a clear indication of the inhibitory activity of Protegrin-2 on SARS-CoV-2 M^pro^, suggesting its potential use against COVID-19. However, further *in vivo* studies are required to validate the *in vitro* results presented here and determine the utility of the peptide as a therapeutic agent against COVID-19.

## 5 Conclusion

In summary, this study identified a potential M^pro^ inhibitor by combining both *in silico* and *in vitro* approaches. This work experimentally validates Protegrin-2 as a potential antiviral drug to treat COVID-19 infection. This research advances the development of peptides-based effective antiviral medicines for COVID-19 infection.

## Ethics approval statement

Ethical approvals were not required for this study.

## Patient consent statement

Not required.

## Permission to reproduce material from other sources

Not required.

## Clinical trial registration

Not required.

## Authorship contribution statement

ZJ, AMG performed experiments, analyzed data, and contributed to writing. PVJ and KB conceptualized, implemented, supervised, analyzed, and wrote the manuscript. All authors read and approved the final manuscript.

## Funding

This work was supported by the College of Health & Life Sciences, Hamad Bin Khalifa University. Research Computing is funded by the 10.13039/100007458Qatar Foundation for Education, Science and Community Development (http://www.qf.org.qa).

## CRediT authorship contribution statement

**Zainab Jan:** Performed experiments, analysed data and contributed to writing. Read and approved the final manuscript. **Anupriya M Geethakumari:** Performed experiments, analysed data and contributed to writing. Read and approved the final manuscript. **Kabir H Biswas:** Conceptualized, implemented, supervised, analysed and wrote the manuscript. Read and approved the final manuscript. **Puthen V Jithesh:** Conceptualized, implemented, supervised, analysed and wrote the manuscript. Read and approved the final manuscript.

## Declaration of Competing Interest

None to declare.

## Data Availability

Publicly available datasets and tools were employed for this study.
